# ﻿Additional four species of *Tatraea* (Leotiomycetes, Helotiales) in Yunnan Province, China

**DOI:** 10.3897/mycokeys.102.112565

**Published:** 2024-02-14

**Authors:** Cui-Jin-Yi Li, Kandawatte Wedaralalage Thilini Chethana, Prapassorn Damrongkool Eungwanichayapant, De-Qun Zhou, Qi Zhao

**Affiliations:** 1 Key Laboratory for Plant Diversity and Biogeography of East Asia, Yunnan Key Laboratory of Fungal Diversity and Green Development, Kunming Institute of Botany, Chinese Academy of Sciences, Kunming, Yunnan 650201, China; 2 School of Science, Mae Fah Luang University, Chiang Rai, 57100, Thailand; 3 Institute of Applied Fungi, Southwest Forestry University, Kunming, Yunnan 650224, China; 4 Center of Excellence in Fungal Research, Mae Fah Luang University, Chiang Rai, 57100, Thailand; 5 Institute of Fanjing Mountain National Park, Tongren University, Tongren, Guizhou 554300, China

**Keywords:** Four new species, Helotiaceae, phylogeny, saprobic fungi, taxonomy

## Abstract

During the investigations of discomycetes in Yunnan, China, five species of *Tatraea* were discovered on decayed, decorticated oak trees or unidentified wood. All species have typical disc-like, large fruiting bodies with grey, brown or greyish-green colors. The ITS sequence analysis showed that they belong to *Tatraea* (Helotiaceae, Helotiales) and the LSU and ITS combination revealed a different topology within the genus. Four species, *T.clepsydriformis*, *T.griseoturcoisina*, *T.yunnanensis* and *T.yuxiensis* were established as new species, and *T.aseptata* was collected and described on oak woods. The pairwise homoplasy index (PHI) test results indicated that there is no significant genetic recombination (Φ*w* = 1.0) between all related species pairs. All the species described here are supported by descriptions, illustrations and multi-gene analyses.

## ﻿Introduction

*Tatraea* Svrček belongs to Helotiaceae, Helotiales, Leotiomycetes (Wijayawardene et al. 2022) and is characterized by large, brown or grey cupulate apothecia with a stipe, distinct dark-colored subhymenium, hyaline medullary excipulum, light-brown colored ectal excipulum, large asci and ascospores (Velenovský 1934; Baral et al. 1999). The type species, *Tatraeadumbirensis* (Velen.) Svrček was initially recognized as *Helotiumdumbirense* in Leotiaceae based on the materials collected in the Nizke Tatra Mountains, Slovakia (Velenovský 1934). In 1985, Svrček re-observed and measured ascospores, and synonymized the species with *Rutstroemiamacrospora* (Peck) Kanouse (Velenovský 1934). However, Spooner (1987, 1988) transferred both *H.dumbirense* and *R.macrospora* to Sclerotiniaceae and synonymized them into *Ciboriapeckiana* (Cooke) Korf and *Ciboriadumbirensis*, respectively, based on their morphological characteristics. After that, Svrček (1993) erected *Tatraea* based on the distinct characters between the materials from Europe and *C.peckiana*. Baral et al. (1999) also accepted the genus *Tatraea* and transferred *Tatraeamacrospora* (Peck) Baral back. However, Baral et al. (1999) did not observe any isodiametric cells in hypothecium, a character significant of Sclerotiniaceae. Recent research showed *Tatraea* belonging to Helotiaceae, and the third species was added by a subsequent study (Vasilyeva 2010). To date, there are three species accepted in *Tatraea* (Index Fungorum 2023).

To date, members of *Tatraea* have been only found as saprobes on the rotting and permanently moist, decorticated trunks of beech wood (*Fagussylvatica*), rarely occurring on *Fraxinusexcelsior* or *Betula* and have been reported in Austria, China, Croatia, Denmark, France, Germany, Great Britain, Italy, Slovakia, Spain, Sweden and Switzerland (Svrček 1993; Baral et al. 1999, 2013a, 2013b; Holec et al. 2015). The significance of fungi in forest ecosystems was reviewed by Niego et al. (2023a), highlighting their diverse functional contributions. This emphasizes the critical need to integrate fungal contributions into ecological conservation policies. Additionally, fungi hold significant value, encompassing not only the economic worth of wild and cultivated mushrooms but also the augmented value derived from fungal products and their involvement in various production processes. Furthermore, fungal involvement in ecosystem processes like carbon sequestration and recreational foraging also increases their traded value. In their study, Niego et al. (2023b) provided estimates to support more effective ecological conservation policies for fungal resources, highlighting the importance of studying and conserving these organisms. Decay fungi are able to produce enzymes that degrade components of wood, such as lignin, cellulose and xylans (Bucher et al. 2004) and are known as lignicolous fungi. Different lignicolous fungi prefer dead wood at different stages of decaying, for example, *Tatraea* mainly grows and decomposes the intermediate and late stages of wood decay (DS4) (Svrček 1993; Baral et al. 1999, 2013a; Heilmann-Clausen 2001; Holec et al. 2015; Dvořák 2017; Ujházy et al. 2018; Kunca et al. 2022). Due to the high density of managed forests, their low understory vegetation diversity compared to that of primary forests as well as the lack of late-stage decayed wood, members of *Tatraea* were rarely discovered in managed forests. Thus *T.dumbirensis* was considered an indicator of the primary forest and forest continuity, but also rarely collected in beech-dominated managed forests (Dvořák 2017; Ujházy et al. 2018). In China, only one species (*Tatraeaaseptata* H.L. Su & Q. Zhao) was discovered in the protected primary forests, and other species were mainly found in the Center of Europe (Baral et al. 1999). *Tatraea* species are mostly found in old, natural primary montane forests (Baral et al. 1999). These species may have specialized adaptations to the undisturbed, virgin primary forests, contributing to overall biodiversity. Hence, they might not thrive or even survive in disturbed or secondary forests. These findings stress the importance of accurate management of primary forests to conserve their fungal diversity as well as the fungal gene pool (Baral et al. 1999). Furthermore, the rarity of these *Tatraea* species also highlights the importance of conducting studies on rare Leotiomycetes fungi to conserve them before they become extinct.

We have been conducting comprehensive studies on discomycetes, encompassing investigations into taxonomy, species diversity and evolutionary research, among other aspects (Ekanayaka et al. 2017, 2018, 2019; Lestari et al. 2022, 2023; Phutthacharoen et al. 2022). In this study, the authors aim to investigate the diversity of discomycetes in Yunnan Province, China. During our exploration, we discovered and collected the rare *Tatraea* species. In this study, we identified four new species of *Tatraea* on decayed and decorticated wood with detailed morphological descriptions and illustrations as recommended by Chethana et al. (2021). In previous studies, the classification of *Tatraea* mainly relied on morphological evidence, and only ITS sequences were available for phylogenetic analysis. Here, we provide additional gene regions and complete morphologies for future taxonomic and evolutionary research.

## ﻿Materials and methods

### ﻿Sample collection and morphological studies

Specimens were collected from decayed wood in Yunnan Province, China, during field investigations conducted from June, 2021 to October, 2022. All samples were obtained from highly humid natural broadleaf forests and protected areas rarely accessed by humans. During the collection period, the temperature of the collection site was basically in the range of 17 °C to 27 °C, and the temperature of Jingdong County, Puer City was 14 °C to 16 °C due to the influence of high altitude. The fruiting bodies were found on the surface of extremely wet decaying wood following rainfall events. The specimens with their substrates were gently wrapped in a single layer of tissue, rotated and pinched ends tightly with a hollow center to prevent squeezing the specimen. The specimens dried naturally in air, re-wrapped in a hard-paper boxes containing a small amount of silica gel and rehydrated before being observed in the laboratory.

Fresh apothecia were photographed in the field by a Canon EOS M100 camera (Canon Co. LTD, Japan). The dried and partially fresh apothecia were captured using a Canon EOS 70D(W) digital camera attached to a C-PSN stereomicroscope (Nikon Corporation Tokyo, Japan). The dried apothecia were sectioned by hand using razor blades and photographed by a charge-coupled device SC 2000C attached to a Nikon ECLIPSE Ni-U compound microscope (Nikon Corporation Tokyo, Japan). Vertical sections were used to observe the excipulum and hymenium. Asci, ascospores and paraphyses were observed by mounting squashed mature apothecia in water. Melzer’s reagent checked the blue iodine reaction of the ascus apex.

All measurements were carried out using Tarosoft (R) Image Framework program (IFW) and modified in Adobe Photoshop 2020 (Adobe system, USA). Q value indicates the length to width ratio of ascospores, n indicates the number of measured structures, and Q_m_ indicates the average of Q value. The size of apothecia was defined as large (greater than 3.5 mm wide), moderate (greater than 2.5 mm and less than 3.5 mm wide) and small (less than 2.5 mm wide) based on mean and extreme values. The length of stipes was defined as long (longer than 1.1 mm), moderate (greater than 0.4 mm and less than 1.1 mm) and short (shorter than 0.4 mm). The colors of apothecia were determined following Kornerup and Wanscher (1967). The dried specimens were deposited at the Herbarium of Cryptogams, Kunming Institute of Botany Academia Sinica (KUN-HKAS). Facesoffungi numbers were obtained as in Jayasiri et al. (2015), and Index Fungorum numbers were obtained as in Index Fungorum (2023). Furthermore, details of all the species described in this study were uploaded to the Discomycetes website (https://discomycetes.org/, Lestari et al. 2023).

### ﻿DNA extraction, PCR amplification, and sequencing

Two to three mature fruit bodies were carefully selected and thoroughly cleaned using sterilized water and 75% alcoholic solution. Subsequently, several layers of epidermal cells were meticulously removed using sterilized surgical blades.Following this step, approximately 1 mm^3^ of tissue was meticulously collected from both the receptacles and stipes using new sterile surgical blades. The collected tissue was then transferred into a sterile 1.5 mL centrifuge tube. Total genomic DNA was extracted using the TriliefTM Plant Genomic DNA Kit (Tsingke Biological Technology Co., LTD, Beijing, China). The total reaction volume for the Polymerase Chain Reaction (PCR) was 25 μl, containing 12.5 μl of 2 × Power Taq PCR MasterMix, 7.5 μl of sterile deionized water, 1 μl of each primer (100 μM stock), and 3 μl of DNA template. The amplifications were performed in a TC-type gene amplifier (LifeECO) (Hangzhou Bori Technology Co., LTD, Hangzhou City, Zhejiang Province, China). The primers used in this study are shown in Table 1. The conditions of PCR for each gene are as follows: for the ITS, LSU, mtSSU and *RPB1*, initial denaturation at 94 °C for 3 min, 35 cycles of denaturation at 94 °C for 30 s, 40 s of annealing at 53 °C, 1 min elongation at 72 °C, followed by a final extension for 10 min at 72 °C; for the *RPB2* initial denaturation at 94 °C for 5 min, 35 cycles of denaturation at 94 °C for 1 min, 1 min of annealing at 56 °C for *RPB2*, 1 min elongation at 72 °C, followed by a final extension for 10 min at 72 °C. The PCR products were verified by 1% agarose gel electrophoresis followed by staining with TS-GelRed Ver.2 10,000 × in Water (Tsingke Biological Technology Co., LTD, Beijing, China). Products were sequenced at Tsingke biological technology Co., LTD, Beijing, China.

**Table 1.** Primers used for the PCR amplifications in this study.

Locus	Primers	Nucleotide sequence (5’- 3’)	Reference
ITS	ITS1-F (F)	5’-TCCGTAGGTGAACCTGCGG-3’	(White et al. 1990)
	ITS4 (R)	5’-TCCTCCGCTTATTGATATGC-3’	
LSU	LR0R (F)	5’-ACCCGCTGAACTTAAGC-3’	(Vilgalys and Hester 1990)
	LR5 (R)	5’-TCCTGAGGGAAACTTCG-3’	
mtSSU	mrSSU1 (F)	5’-AGCAGTGAGGAATATTGGTC-3’	(Zoller et al. 1999)
	mrSSU3R (R)	5’-ATGTGGCACGTCTATAGCCC-3’	
*RPB1*	RPB1-Af (F)	5’-GARTGYCCDGGDCAYTTYGG-3’	(Stiller and Hall 1997)
	RPB1-Cr (R)	5’-CCNGCDATNTCRTTRTCCATRTA-3’	
*RPB2*	fRPB2-5F (F)	5’-GAYGAYMGWGATCAYTTYGG-3’	(Liu et al. 1999)
	fRPB2-7cR (R)	5’-CCCATWGCYTGCTTMCCCAT-3’	

### ﻿Sequence assembly and alignment

Sequences were assembled in ContigExpress (Invitrogen, USA), and then checked and edited in BioEdit 7.2.5.0 (Hall 1999). The homologous sequences were selected based on the results of the BLASTn search performed against the GenBank database available at NCBI. All new and related sequences used in this study were derived from GenBank and used for phylogenetic analyses. Two species in *Chlorociboria* (Chlorociboriaceae, Helotiales) were selected as the outgroup taxa.

**Figure 1.**
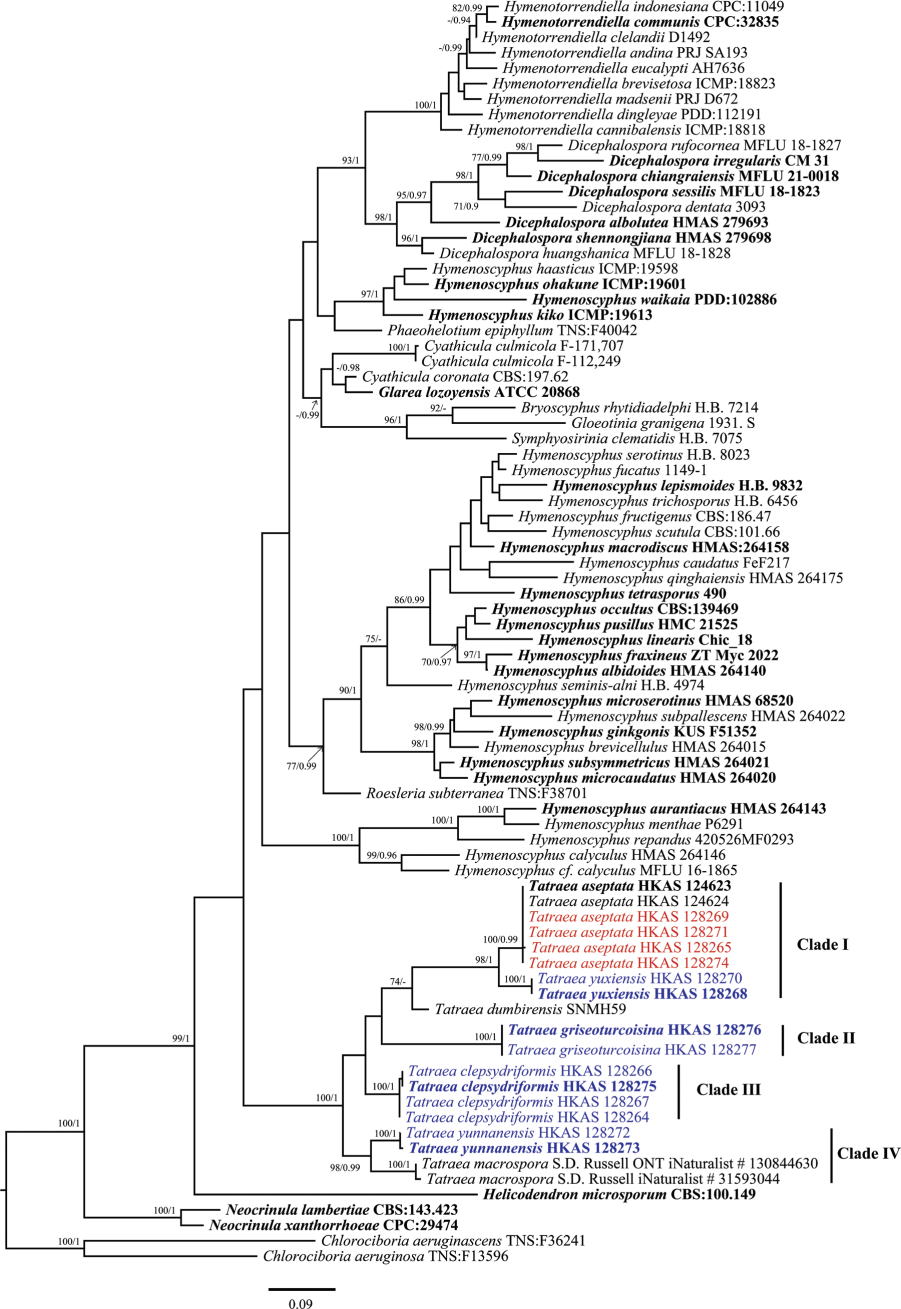
Maximum likelihood tree based on the ITS sequence, showing the phylogenetic position of *Tatraea*. The ML bootstrap proportions (ML-BP) equal to or higher than 70% and Bayesian posterior proportions (BI-PP) equal to or higher than 0.90 are shown near the branches of the phylogenetic tree. Newly generated isolates of the current study are shown in blue and ex-types in bold.

**Figure 2.**
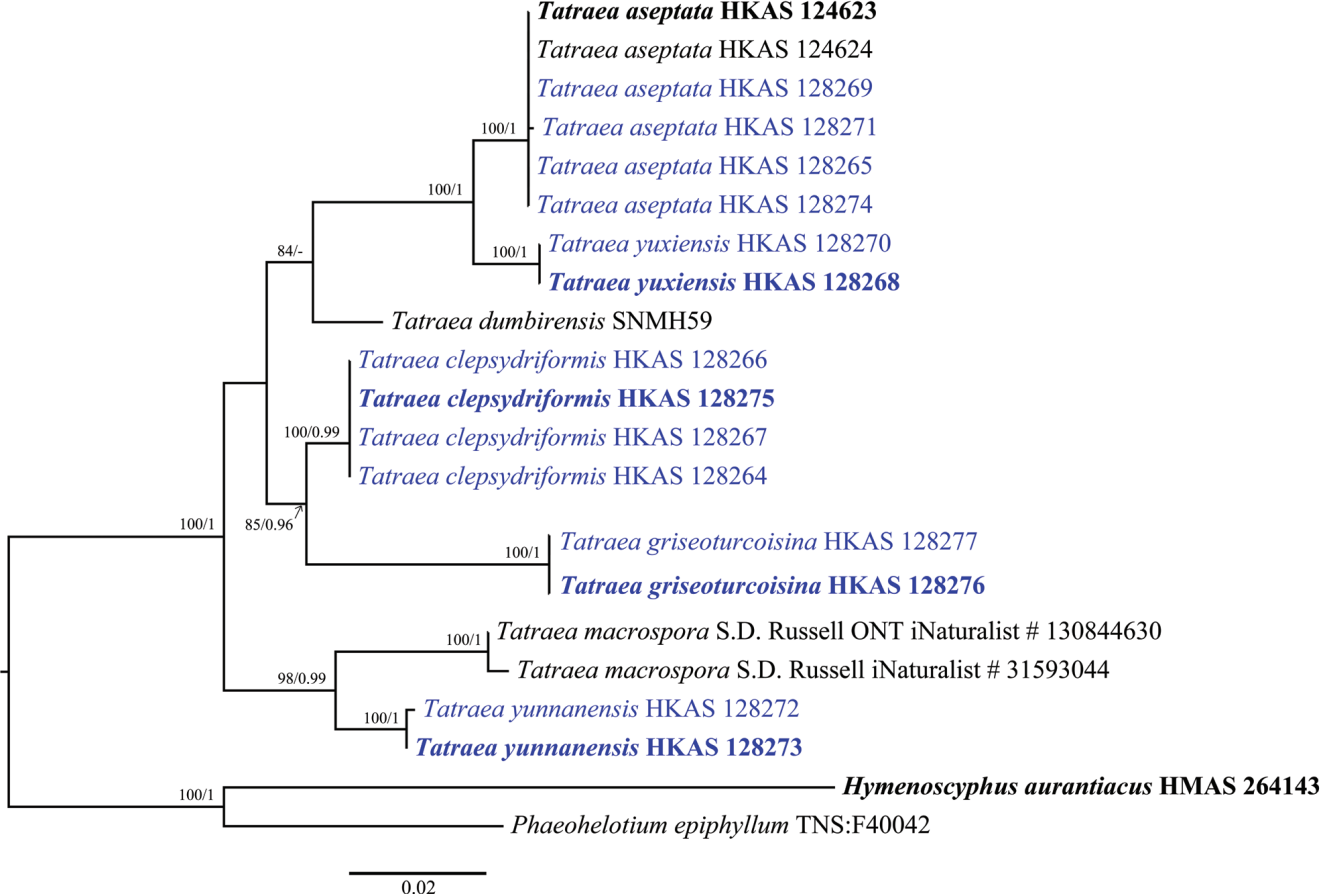
Maximum likelihood tree based on a combined dataset of LSU and ITS sequences for the genus *Tatraea*. The ML bootstrap proportions (ML-BP) equal to or higher than 70% and Bayesian posterior proportions (BI-PP) equal to or higher than 0.90 are shown near the branches on the phylogenetic tree. Newly generated isolates of the current study are shown in blue and ex-types are shown in bold.

**Figure 3.**
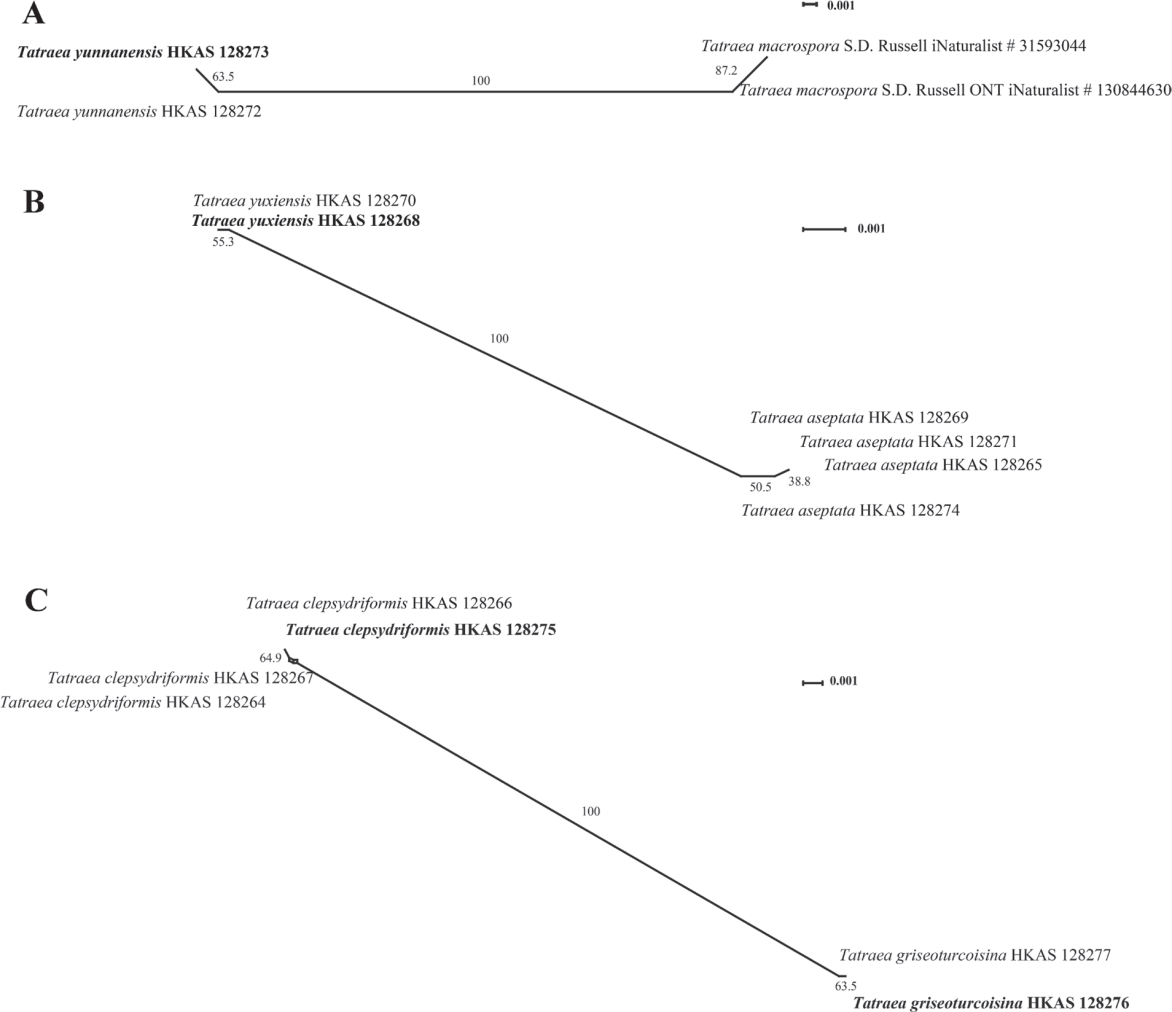
The results of the pairwise homoplasy index (PHI) test for the closely related species in *Tatraea* using LogDet transformation. A. PHI test for *Tatraeayunnanensis* vs. *Tatraeamacrospora*. B. PHI test for *Tatraeayuxiensis vs. Tatraeaaseptata*. C. PHI test for T*atraea clepsydriformis vs. Tatraeagriseoturcoisina*. PHI test results (*Φw* < 0.05) indicate significant recombination within the dataset.

The datasets were aligned in MAFFT v. 7 (Katoh et al. 2019) with G-INS-i as the iterative refinement and default parameters were applied except for the gap penalty, which was changed to 1.00, and improved manually in BioEdit v. 7.2.5.0. Then, datasets were trimmed in TrimAl v.1.3 using the gappyout option (Capella-Gutiérrez et al. 2009). The multiple loci association matrixes were concatenated to a combined dataset in SequenceMatrix 1.7.8. (Vaidya et al. 2011). Due to the lack of sequence data for protein genes, the phylogenetic tree was constructed using the ITS gene and the combined LSU and ITS gene regions (Figs 1, 2). The combined ITS, LSU, mtSSU, *RPB1* and *RPB2* dataset was used to analyze the recombination level within phylogenetically and closely related species (Fig. 3). The ALTER (Alignment Transformation EnviRonment) online tool was used to convert from “.fasta” to “.nexus” format. The newly generated sequences in this study were deposited in GenBank (Table 2), and the combined alignment was deposited at the TreeBASE (submission ID: 30884).

**Table 2.** Detailed information and corresponding GenBank accession numbers of the taxa used in the phylogenetic analyses of this study. ‘^†^’ Denotes type species. Newly generated sequences are shown in bold. ‘-’: indicates sequence data is not available.

Taxon name	Voucher	Gene accession No.				
		ITS	LSU	mtSSU	*RPB1*	*RPB2*
*Bryoscyphusrhytidiadelphi*	H.B. 7214	OM808923	–	–	–	–
*Chlorociboriaaeruginosa*	TNS-F-13596	LC425047	–	–	–	–
*C.aeruginascens*	TNS-F-36241	LC425045	–	–	–	–
*Cyathiculacoronata*	CBS:197.62	MH858141	–	–	–	–
*Cy.culmicola*	F-171,707	FJ005119	–	–	–	–
*Cy.culmicola*	F-112,249	FJ005121	–	–	–	–
*Dicephalosporaalbolutea* ^†^	HMAS 279693	MK425601	–	–	–	–
*D.chiangraiensis* ^†^	MFLU 21-0018	MZ241817	–	–	–	–
*D.dentata*	3093	KP204263	–	–	–	–
*D.huangshanica*	MFLU 18-1828	MK584979	–	–	–	–
*D.irregularis* ^†^	CM 31	ON511117	–	–	–	–
*D.rufocornea*	MFLU 18-1827	MK584978	–	–	–	–
*D.sessilis* ^†^	MFLU 18-1823	NR_163779	–	–	–	–
*D.shennongjiana* ^†^	HMAS 279698	MK425606	–	–	–	–
*Glarealozoyensis* ^†^	ATCC 20868	NR_137138	–	–	–	–
*Gloeotiniagranigena*	1931. S	Z81432	–	–	–	–
*Helicodendronmicrosporum* ^†^	CBS:100.149	NR_137974	–	–	–	–
*Hymenoscyphusalbidoides* ^†^	HMAS 264140	NR_154903	–	–	–	–
*H.aurantiacus* ^†^	HMAS 264143	NR_154907	NG_059509	–	–	–
*H.brevicellulus*	HMAS 264015	JX977149	–	–	–	–
*H.calyculus*	HMAS 264146	KJ472291	–	–	–	–
*H.caudatus*	FeF217	MZ492984	–	–	–	–
*H.cf.calyculus*	MFLU 16-1865	MK584966	–	–	–	–
*H.fraxineus* ^†^	ZT Myc 2022	NR_111479	–	–	–	–
*H.fructigenus*	CBS:186.47	MH856211	–	–	–	–
*H.fucatus*	1149-1	MW959791	–	–	–	–
*H.ginkgonis* ^†^	KUS F51352	NR_119669	–	–	–	–
*H.kiko* ^†^	ICMP:19613	NR_137110	–	–	–	–
*H.lepismoides* ^†^	H.B. 9832	KM199777	–	–	–	–
*H.linearis* ^†^	Chic_18	KM114535	–	–	–	–
*H.macrodiscus* ^†^	HMAS 264158	NR_154908	–	–	–	–
*H.menthae*	P6291	MH063781	–	–	–	–
*H.microcaudatus* ^†^	HMAS 264020	JX977156	–	–	–	–
*H.microserotinus* ^†^	HMAS 68520	NR_132814	–	–	–	–
*H.occultus* ^†^	CBS:139.469	NR_147434	–	–	–	–
*H.ohakune* ^†^	ICMP:19601	NR_137109	–	–	–	–
*H.pusillus* ^†^	HMC 21525	MH476516	–	–	–	–
*H.repandus*	420526MF0293	MG712335	–	–	–	–
*H.scutula*	CBS:101.66	MH858736	–	–	–	–
*H.seminis-alni*	H.B. 4974	KM114536	–	–	–	–
*H.serotinus*	H.B. 8023	KM114541	–	–	–	–
*H.subpallescens*	HMAS 264022	JX977154	–	–	–	–
*H.subsymmetricus* ^†^	HMAS 264021	JX977153	–	–	–	–
*H.tetrasporus* ^†^	490	KJ472302	–	–	–	–
*H.trichosporus*	H.B. 6456	KM114538	–	–	–	–
*H.waikaia* ^†^	PDD:102886	NR_137111	–	–	–	–
*Hymenotorrendiellaandina*	PRJ SA193	KJ606682	–	–	–	–
*Hy.brevisetosa*	ICMP:18823	JN225946	–	–	–	–
*Hy.cannibalensis*	ICMP:18818	JN225947	–	–	–	–
*Hy.clelandii*	D1492	OK346623	–	–	–	–
*Hy.communis* ^†^	CPC:32835	NR_170836	–	–	–	–
*Hy.dingleyae*	PDD:112191	MK039692	–	–	–	–
*Hy.eucalypti*	AH7636	KF588379	–	–	–	–
*Hy.indonesiana*	CPC:11049	DQ195787	–	–	–	–
*Hy.madsenii*	PRJ:D672	AY755336	–	–	–	–
*Neocrinulalambertiae* ^†^	CBS:143.423	NR_156388	–	–	–	–
*N.xanthorrhoeae* ^†^	CPC:29474	NR_154252	–	–	–	–
*Phaeohelotiumepiphyllum*	TNS:F40042	AB926061	AB926130			
*Roesleriasubterranea*	TNS:F38701	AB628057	–	–	–	–
*Symphyosiriniaclematidis*	H.B. 7075	OM808922	–	–	–	–
*Tatraeadumbirensis*	SNMH59	MK907417	–	–	–	–
*T.macrospora*	S.D. Russell ONT iNaturalist # 130844630	OP643029	–	–	–	–
*T.macrospora*	S.D. Russell iNaturalist # 31593044	OM473784	–	–	–	–
*T.aseptata*	HKAS 124624	OP538031	–	–	–	–
*T.aseptata* ^†^	HKAS 124623	OP538030	–	–	–	–
***T.aseptata***	HKAS 128269	**OQ921780**	**OR214956**	–	**OR703635**	–
***T.aseptata***	HKAS 128274	**OQ921783**	**OR214952**	**OR237204**	**OR703633**	**OR735340**
***T.aseptata***	HKAS 128271	**OQ921782**	**OR220038**	**OR237210**	**OR703636**	**OR735342**
***T.aseptata***	HKAS 128265	**OQ921777**	**OR214955**	**OR237207**	**OR703634**	**OR735341**
***T.clepsydriformis***	HKAS 128266	**OQ520277**	**OR214945**	**OR271555**	**OR703642**	**OR735348**
***T.clepsydriformis* ^†^**	HKAS 128275	**OQ520268**	**OR214946**	**OR237205**	**OR703641**	**OR735347**
***T.clepsydriformis***	HKAS 128264	**OQ921768**	**OR214949**	**OR237203**	**OR703643**	–
***T.clepsydriformis***	HKAS 128267	**OQ921773**	**OR214951**	**OR271554**	**OR703644**	**OR735349**
***T.griseoturcoisina* ^†^**	HKAS 128276	**OQ520299**	**OR214959**	**OR237211**	**OR703646**	**OR735351**
***T.griseoturcoisina***	HKAS 128277	**OQ520298**	**OR214965**	**OR237207**	**OR703645**	**OR735350**
***T.yunnanensis***	HKAS 128272	**OQ546436**	**OR220043**	**OR237209**	**OR703639**	**OR735345**
***T.yunnanensis* ^†^**	HKAS 128273	**OQ520294**	**OR220044**	**OR237212**	**OR703640**	**OR735346**
***T.yuxiensis* ^†^**	HKAS 128268	**OQ546437**	**OR220042**	**OR237202**	**OR703637**	**OR735343**
***T.yuxiensis***	HKAS 128270	**OQ546435**	**OR220039**	**OR237208**	**OR703638**	**OR735344**

### ﻿Phylogenetic analyses

Maximum likelihood (ML) analysis was performed in RAxML-HPC2 on XSEDE (8.2.12) on the CIPRES Science Gateway platform (http://www.phylo.org/portal2) using the GTR model with 1,000 bootstrap replications. Bayesian inference (BI) analysis was performed using MrBayes v. 3.1.2. The Markov Chain Monte Carlo sampling (MCMC) was used to evaluate the posterior probabilities (PP). The general time-reversible model with a discrete gamma distribution coupled with a proportion of an invariant (GTR+I+G) was selected for nLSU and ITS as the best model using MrModeltest v.2.3 (Nylander et al. 2004). Four simultaneous Markov Chains were run for 2,000,000 generations, with trees sampling at every 100^th^ generation. The 25% of the trees representing the burn-in phase were discarded, and the remaining trees were used to calculate the posterior probability. The finalized phylogenetic tree was visualized in Figtree v.1.4.0 (Rambaut 2012) and edited in Adobe Illustrator 2020 and Adobe Photoshop 2020 (https://www.adobe.com/). Splitstree4 4.17.1 was used to determine the recombination level between phylogenetically and closely related but ambiguous species based on the PHI (pairwise homoplasy index) value (Taylor et al. 2000; Silvestro and Michalak 2012). The relationships between the two species were shown in splits graphs with the Log-Det transformation option. The relationship between *T.macrospora* and *T.yunnanensis* was visualized by constructing a split graph (Fig. 3A) from ITS. The relationship between the other two pairs (*T.yuxiensis* and *T.aseptata*, and *T.griseoturcoisina* and *T.clepsydriformis*) were visualized by constructing splits graphs, Fig. 3B and Fig. 3C, respectively, from 5-locus concatenated dataset. A pairwise homoplasy index below a 0.05 threshold (*Φw* < 0.05) indicates the presence of significant recombination between the two species (Chethana et al. 2017).

## ﻿Results

### ﻿Phylogenetic analyses

The dataset for the phylogenetic analysis based on the ITS gene consists of 69 taxa, represented by 81 isolates, including two outgroup taxa, *Chlorociboriaaeruginosa* (TNS:F13596) and *Chlorociboriaaeruginascens* (TNS:F36241). The dataset contains 550 total characters with gaps. The combined alignment contains 239 constant characters, 54 variable and parsimony uninformative characters and 254 parsimony-informative characters. The RAxML analysis of the ITS gene dataset yielded the best-scoring tree with a final likelihood value of -8336.600892 (Fig. 1). The maximum likelihood matrix comprises 366 distinct alignment patterns with 8.85% undetermined characters or gaps. Estimated base frequencies are as follows: A = 0.219317, C = 0.265186, G = 0.257196, T = 0.258302; substitution rates AC = 1.749614, AG = 2.352229, AT = 1.537478, CG = 0.647334, CT = 5.060114, GT = 1.000000; gamma distribution shape parameter α = 0.360268. The LSU and ITS concatenated dataset consists of 9 taxa, represented by 21 isolates, including two outgroup taxa, *Hymenoscyphusaurantiacus* (HMAS 264143) and *Phaeohelotiumepiphyllum* (TNS:F40042). The concatenated dataset contains 1744 aligned nucleotide sites, including 1256 bp for the LSU region and 488 bp for the ITS region with gaps. The combined alignment contains 1471 constant characters, 89 variable and parsimony uninformative characters and 184 parsimony-informative characters. The RAxML analysis of the combined dataset (LSU and ITS) yielded the best-scoring tree with a final likelihood value of -4243.290550 (Fig. 2). The dataset comprises 259 distinct alignment patterns with 31.66% undetermined characters or gaps. Estimated base frequencies are as follows: A = 0.227902, C = 0.244223, G = 0.295968, T = 0.231906; substitution rates AC = 0.961500, AG = 2.058057, AT = 0.608426, CG = 0.460988, CT = 7.662978, GT = 1.000000; gamma distribution shape parameter α = 0.183205. Species in *Hymenoscyphus* showed different topologies in ML and BI analyses, but the support values for the nodes are less. Despite the different taxon sampling, the topological structure of the phylogenetic tree shown in Fig. 1 is similar to that of Johnston et al. (2019). In the ML and Bayes analyses, *Tatraea* formed a monophyletic clade within Helotiaceae with 67% ML bootstrap support and 0.98 Bayesian probability in the ITS phylogeny (Fig. 1). Some nodes in Clade II and Clade III have low support values (Fig. 1). In Clade I, our collections of *Tatraeaaseptata* clustered with the type species and formed a sub-clade sister to *T.yuxiensis* with 98% ML support and 1.00 Bayesian probability support. However, this clade comprising *T.aseptata* and *T.yuxiensis* separated from *T.dumbirensis* with 74% ML bootstrap support and 0.57 Bayesian probability. Collections of *T.clepsydriformis* and *T.griseoturcoisina* formed two individual clades in Fig. 1 with 50% ML bootstrap support and 0.80 Bayesian probability, and 52% ML bootstrap support and 0.92 Bayesian probability, respectively. However, these two species clustered as a separate sub-clade with 85% ML support and 0.96 Bayesian probability support in the ITS and LSU combined phylogeny (Fig. 2). *Tatraeayunnanensis* clustered with *T.macrospora* with 98% ML bootstrap support and 0.99 Bayesian probability support in the LSU and ITS phylogeny (Fig. 2). A pairwise homoplasy index below 0.05 typically indicated the presence of significant recombination among the groups. In our analysis, the pairwise homoplasy index (PHI or *Φw*) for three pairs of species (*T.macrospora vs. T.yunnanensis*, *T.yuxiensis vs. T*. *aseptata* and *T.griseoturcoisina vs. T.clepsydriformis*) were 1.0, 1.0 and 0.4185, respectively. These results indicated no significant recombination among these pairs.

### ﻿Taxonomy

#### ﻿ Tatraea aseptata

Taxon classificationFungiHelotialesHelotiaceae

H.L. Su & Q. Zhao

6F1E3758-46B1-5E19-8987-33CD8EE1673C

Index Fungorum: IF559987

Facesoffungi Number: FoF12892

Fig. 4

##### Type material.

***Holotype*.** HKAS 124623.

##### Description.

Saprobic on the decayed branches of oak trees. ***Sexual morph***: Apothecia 2.5–4.7 mm wide (x̄ = 3.3 ± 0.5 mm, n = 27) when fresh, 1–2.4 mm wide × 0.6–1.2 mm high (x̄ = 1.6 ± 0.3 × 0.9 ± 0.1 mm, n = 28) when dry, scattered or gregarious, superficial, discoid with glabrous, short stipe. Disc flat and circular, light brown (7D5–7D6) in wet habitat, slightly dark alabaster grey (5B2) in slightly dried habitat when fresh, edge undulating and slightly curl inward towards the disc, dark brown (8F5–8F6) to dull green (30E4) or greyish green (30E5) when dry, sometimes orange white to pale greenish white (29A2) or dull yellow (3B4) to greyish yellow (3B5–3B6) near center. Margins white when immature and fresh, concolorous to the disc when mature and fresh, white to pale yellow or concolorous to the receptacles when dry. Receptacle smooth and brown (6D7–6D8) when fresh, yellowish brown (5E5–5E6), flank darker when dry, rough and fine pustules on the surface. Stipe 280–725 μm wide × 340–735 μm long (x̄ = 500 ± 148 × 540 ± 125 μm, n = 11), short, broad at upside part, narrower at lower part, golden brown (5D7) when fresh, light brown when dry, finely granular pustules, ridged at maturity. Hymenium 142–190 μm (x̄ = 160 ± 14 μm, n = 15), hyaline. Subhymenium 35–52 μm (x̄ = 44 ± 4 μm, n = 25), dense brown hyphae forming a *textura intricata*, hyphae 3.4–4.3 μm (x̄ = 3.8 ± 0.3 μm, n = 25) diam., gather with excipulum at the margin. Medullary excipulum 120–145 μm (x̄ = 133 ± 7 μm, n = 15), thick, well-developed, comprised of thin-walled, septate, pale brown and slightly loose hyphae of *textura intricata*, hyphae 4.3–7.4 μm (x̄ = 6.0 ± 0.8 μm, n = 25) diam., hyaline, becoming dense and well-organized, parallel near to the ectal excipulum, non-gelatinous. Ectal excipulum visible, different from the medullary excipulum, the inner layers generally consists of 3–4 layers *textura globulosa* to *textura angularis* cells, 30–48 μm (x̄ = 38 ± 4 μm, n = 35) thick, moderately thick-walled, cells 8.4–16.5 μm (x̄ = 13.0 ± 1.9 μm, n = 50) diam., wall 0.63–1.54 μm (x̄ = 1.01 ± 0.18 μm, n = 70) thick; the outer layers partially uneven proliferous to 8–12 layers, stack into triangles to trapezoids, 50–89 μm (x̄ = 69 ± 9 μm, n = 60) thick (including the inner layers), cells 3–11 μm (x̄ = 8 ± 1.7 μm, n = 75) diam., wall 0.6–1.7 μm (x̄ = 0.95 ± 0.21 μm, n = 65) thick; cells from the outer to the inner layers gradually increase in diameter, brown to colorless; terminal cells of 3–4 layers at flank stretch to 13.1–15.3 μm long × 2.6–3.8 μm wide (x̄ = 14.1 ± 0.8 × 3.3 ± 0.5 mm, n = 10), straight, ends narrow and slightly sharp, thin-walled, brown. Paraphyses 1.9–3.7 μm (x̄ = 2.6 ± 0.4 μm, n = 50) wide, hyaline, filiform, rounded apex, 0–2-septate, unbranched, with conspicuous lipid bodies, scarcely extending beyond the asci. Asci (132–)136.7–157.8(–172) × (12.5–)13.2–16.0(–17.3) μm (x̄ = 148.2 ± 7.8 × 14.5 ± 1.0 μm, n = 40), unitunicate, 8-spored, almost filling the whole asci, clavate, slightly curved, apically rounded with an amyloid apical pore in Melzer’s reagent, an incrassated wall at apex, 7.4–11.1 μm wide × 3.7–6.1 μm high (x̄ = 9.0 ± 0.7 × 4.8 ± 0.5 μm, n = 40), slightly constricted downward, tapering to obconical or short subtruncated base, sometimes not obvious, croziers present. Ascospore (21.8–)24.6–31.6(–33.7) × (7.6–)7.8–10.0(–10.8) μm (x̄ = 27.4 ± 2.3 × 8.8 ± 0.7 μm, n = 80), Q = (2.3–)2.5–3.7(–4.0), Qm = 3.1 ± 0.3, overlapping uniseriate, slightly asymmetrical, reniform with a large guttule and several multiple granules, obtusely rounded at the apex, slightly pointed at the base, hyaline, thin-walled, smooth and aseptate. ***Asexual morph***: Undetermined.

**Figure 4.**
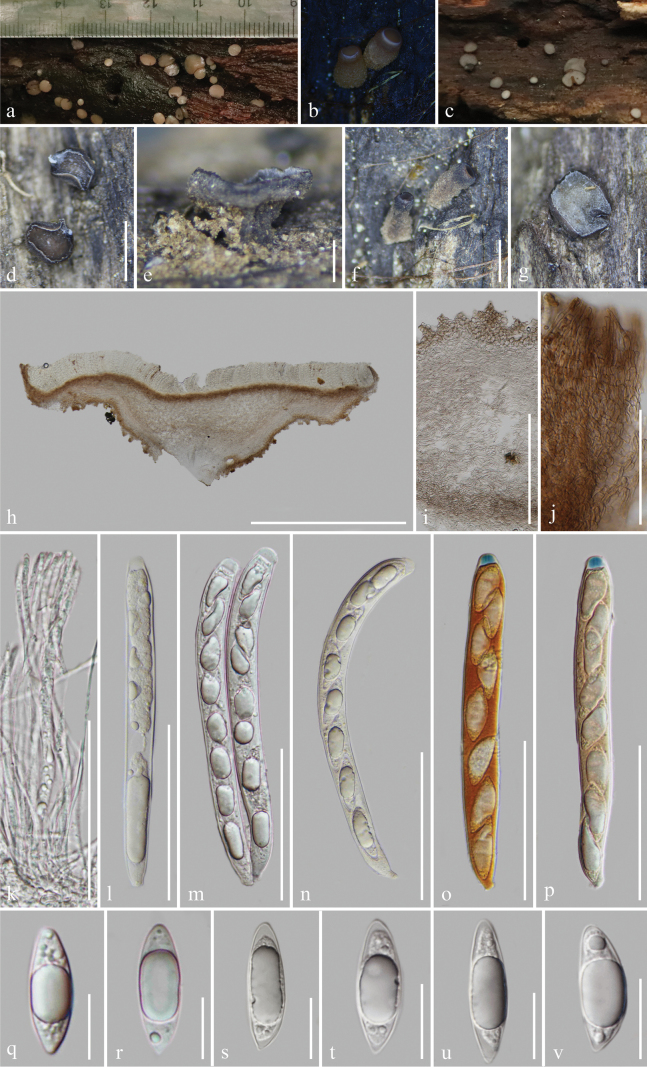
*Tatraeaaseptata* (HKAS 128275) **a–c** Fresh ascomata on the wood **d–g** dried ascomata on the wood **h** vertical section of an ascoma **i–j** excipulum **k** paraphyses **l–p** asci (**o–p** asci in Meltzer’s reagent) **q–v** ascospores. Scale bars: 1.5 mm (**d**); 400 μm (**e**); 800 μm (**f–g**); 1000 μm (**h**); 200 μm (**i**); 100 μm (**j**); 70 μm (**k–p**); 15 μm (**q–v**).

##### Material examined.

China, Yunnan Province, Puer City, Jingdong County, altitude 2455 m, on the decayed oak tree twig, 23 August 2022, Cuijinyi Li, LCJY-1221 (HKAS 128274); *ibid.*, Ailao Mountain, altitude 2520 m, on mossy, decaying unknown wood, 9 June 2022, Cuijinyi Li, LCJY-743 (HKAS 128271); *ibid.*, Kunming City, Panlong District, altitude 1920 m, on the decayed oak tree twig, 25 May 2022, Cuijinyi Li, LCJY-477 (HKAS 128265); *ibid.*, Yuxi City, Xinping County, altitude 1920 m, on soft decayed unknown wood, 5 June 2022, Cuijinyi Li, LCJY-601 (HKAS 128269).

##### Notes.

Our collections are clustered with *T.aseptata* H.L. Su & Q. Zhao with 100% ML bootstrap support and 1.0 Bayesian probability. Fruiting bodies are mostly founded on decayed oak tree branches and share similar characteristics with *T.aseptata* by having fresh apothecia of similar size, brown receptacles when dry and the same reniform ascospores. In contrast, our collection also showed differences in their outer ectal excipulum comprising 8–12 layers of uneven proliferous cells with no hairs, thinner medullary excipulum and shorter asci (136.7–157.8 μm vs. 150–185 μm).

#### ﻿ Tatraea clepsydriformis

Taxon classificationFungiHelotialesHelotiaceae

C.J.Y. Li & Q. Zhao sp. nov.

6D2F5518-CCF7-51E3-9C48-26D5B2FFF322

Index Fungorum: IF901178

Facesoffungi Number: Fo15190

Fig. 5

##### Etymology.

The specific epithet refers to the hourglass shape apothecia.

##### Holotype.

HKAS 128275.

##### Description.

Saprobic on the decayed branches of oak tree. ***Sexual morph***: Apothecia 1.3–3.5 mm wide (x̄ = 2.5 ± 0.7 mm, n = 13) when fresh, 0.9–1.3 mm wide × 0.6–0.9 mm high (x̄ = 1.1 ± 0.15 × 0.7 ± 0.12 mm, n = 13) when dry, gregarious, superficial, hourglass shape or cupulate, glabrous, with a wide stipe. Disc flat and circular, pale grey (5C1) when fresh, edge slightly curl inward towards the disc, melon yellow (5A6) to apricot yellow (5B6) or pale orange (5A2) near the center, darken to concolorous as receptacle near the edge when dry, or dark blue (20E7) when immature. Margins concolorous to the disc when fresh, white, smooth or dentate when dry. Receptacle slightly rough and dark yellowish brown (5D8) when fresh, slightly rough, light brown (6D8) to hazel brown (6E8) when mature and dry, sometimes edge with white narrow-band, smooth and dark blackish blue (20F8) when immature and dry. Stipe 360–596 μm wide × 463–571 μm long (x̄ = 470 ± 85 × 515 ± 47 μm, n = 13), short and broad, concolorous to the receptacle or pale yellow when fresh, concolorous to dried receptacle when mature, butter yellow (4A5) when immature, slightly rough on surface. Hymenium 122–155 μm (x̄ = 135 ± 12 μm, n = 30), hyaline. Subhymenium (24–)36–60(–65) μm (x̄ = 44 ± 8 μm, n = 37), dense golden brown (5D7) hyphae, forming *textura intricata*, hyphae 2.2–2.9 μm (x̄ = 2.6 ± 0.2 μm, n = 25) wide. Medullary excipulum 335–535 μm (x̄ = 415 ± 51 μm, n = 15) thick, well-developed, comprised of thin-walled, septate, branched, pale brown and slightly loose hyphae of *textura intricata* in center, hyphae 3.3–5.1 μm (x̄ = 4.2 ± 0.5 μm, n = 45) diam., hyaline, near the ectal excipulum becoming well-organized parallel, non-gelatinous. Ectal excipulum 29–80 μm (x̄ = 50 ± 14 μm, n = 48) thick, comprised of 3–5 layers, large cells inside and several outer layers of smaller cells of *textura angularis*, 4.9–15.3 μm (x̄ = 8.9 ± 2.4 μm, n = 64) diam., wall moderately thick, 0.5–1.1 μm (x̄ = 0.7 ± 0.1 μm, n = 52) thick, pale brown to pale yellow from the outer inward the inner layers; proliferous cells not observed; terminal cells at margin inconspicuous elongated. Paraphyses 2.1–3.4 μm (x̄ = 2.6 ± 0.3 μm, n = 45) wide, hyaline, straight and filiform, apically round, 1–3-septate, unbranched, no conspicuous contents, scarcely extending beyond the asci. Asci (104.0–)112.4–135.8 × 8.2–12.2 μm (x̄ = 121.5 ± 5.7 × 10.1 ± 0.9 μm, n = 40), unitunicate, 8-spored, cylindric or subclavate, apically rounded with an amyloid apical pore in Melzer’s reagent, apical wall incrassated, 5.0–8.5 μm wide × 2.0–3.7(–4.4) μm high (x̄ = 6.8 ± 0.7 × 3.1 ± 0.5 μm, n = 40), slightly constricted downward when immature, tapering to a cylindric and aporhynchous, subtruncated base, croziers present. Ascospore (12.9–)14.0–17.9 × 5.1–6.8 μm (x̄ = 15.2 ± 0.9 × 5.7 ± 0.4 μm, n = 65), Q = 2.1–3.2, Qm = 2.7 ± 0.1, overlapping uniseriate, ellipsoidal with a large guttule, obtusely rounded at both ends, slightly pointed at the base, hyaline, almost symmetrical, thin-walled, smooth and aseptate. ***Asexual morph***: Undetermined.

**Figure 5.**
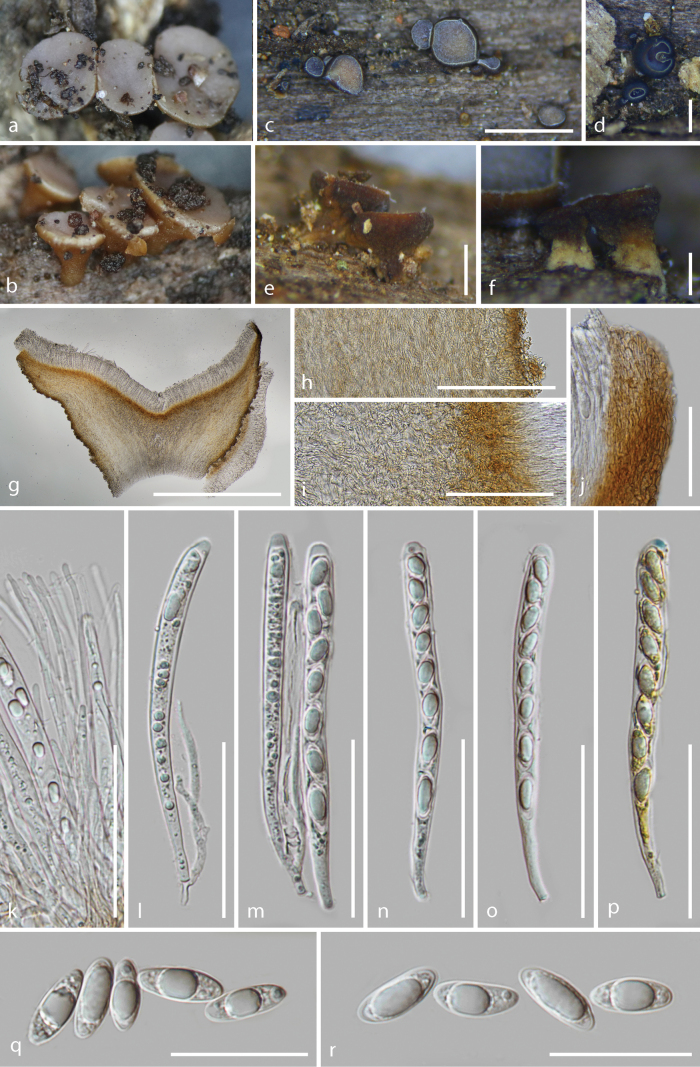
*Tatraeaclepsydriformis* (HKAS 128275, holotype) **a–b** fresh ascomata on the wood **c–f** dried ascomata on the wood **g** vertical section of an ascoma **h–j** excipulum **k** paraphyses **l–p** asci (**o–p** asci in Meltzer’s reagent) **q–r** ascospores. Scale bars: 2 mm (**c**); 600 μm (**d**); 700 μm (**e**); 250 μm (**f**); 800 μm (**g**); 150 μm (**h, j**); 100 μm (**i**); 60 μm (**k–p**); 40 μm (**q–r**).

##### Material examined.

China, Yunnan Province, Puer City, Jingdong County, altitude 2455 m, on the decayed oak tree twig, 23 August 2022, Cuijinyi Li, LCJY-1226 (HKAS 128275, holotype); *ibid.*, Kunming City, Panlong District, altitude 1920 m, on the decayed oak tree twig with ant nests, 29 May 2022, Cuijinyi Li, LCJY-497 (HKAS 128266, paratype); *ibid.*, Yeya Lake, altitude 1900 m, on the decayed oak tree twig with ant nests, 3 July 2021, Cuijinyi Li, LCJY-127 (HKAS 128264, paratype); *ibid.*, Sanjian Mountain, altitude 1950 m, on decayed wood, 18 December 2021, Cuijinyi Li, LCJY-392 (HKAS 128267, paratype).

##### Notes.

The distinctive characteristics of *T.clepsydriformis* are moderate-sized apothecia (2.5 mm wide when fresh), with fresh brown receptacles and stipes, light brown to hazel brown at dry condition, stipes concolorous to receptacles, pale yellow, proliferous cells of ectal excipulum not observed, aporhynchous asci and small, ellipsoidal ascospores without septa.

Phylogenetically, our collections clustered with *T.griseoturcoisina* with 85% ML bootstrap support and 0.96 Bayesian probability in the combined LSU and ITS phylogeny (Fig. 2). Morphologically, both species have small ascospores shown in Suppl. material 1 (shorter than 23 μm). *Tatraeaclepsydriformis* are distinguished from other species by their shorter asci and smaller ascospores except for *T.griseoturcoisina* (Suppl. material 1). *Tatraeaclepsydriformis* differs from *T.griseoturcoisina* by having brown receptacles, a broader medullary excipulum (335–535 μm vs. 164–308 μm) and shorter ascospores (15.2 × 5.7 μm vs. 17.1 × 5.4 μm).

#### ﻿ Tatraea griseoturcoisina

Taxon classificationFungiHelotialesHelotiaceae

C.J.Y. Li & Q. Zhao sp. nov.

95BF68FE-5E53-5D45-AFF5-7158EF477E07

Index Fungorum: IF901179

Facesoffungi Number: Fo15191

Fig. 6

##### Etymology.

The specific epithet refers to the greyish turquoise color of the disc.

##### Holotype.

HKAS 128276.

##### Description.

Saprobic on decayed branches. ***Sexual morph***: Apothecia 2.5–4.0 mm wide (x̄ = 3.1 ± 0.4 mm, n = 27) when fresh, 1.0–2.1 mm wide × 0.6–0.8 mm high (x̄ = 1.6 ± 0.3 × 0.7 ± 0.1 mm, n = 20) when dry, scattered or gregarious, superficial, discoid with thin and short stipitate, glabrous. Disc flat and circular, greyish turquoise (24E5) when fresh in wet habitat, slightly concave in the center, edge slightly curved upwards, deep green (28E8) with greyish green (28D5) to dark greyish green (28F7) when fresh in slightly dried habitat, edge slightly fold inward towards the discs, yellowish white (1A2) to snow white (1A1) when dry. Margins concolorous to the discs when fresh in wet habitat, white when dry or living in the dried habitat. Receptacle not observed when fresh, rough and finely pustules, dark brown to nearly black when dry, with some slightly dark and irregular veins on the surface. Stipe 330–360 μm wide × 220–440 μm long (x̄ = 350 ± 89 × 330 ± 8 μm, n = 5), short and thin, rough and finely pustules, concolorous to the receptacle. Hymenium 103–138 μm (x̄ = 117 ± 8 μm, n = 25), hyaline. Subhymenium 43–66 μm (x̄ = 53 ± 6 μm, n = 15), dense brownish-orange (6C8) hyphae of *textura intricata*, hyphae 1.3–2.9(–3.9) μm (x̄ = 2.2 ± 0.5 μm, n = 50) diam., appear with excipulum at the margin, non-gelatinous. Medullary excipulum 164–308 μm (x̄ = 238 ± 33 μm, n = 15) thick, well-developed, comprised of thin-walled, septate, pale brown to pale yellow cells of *textura intricata*, hyphae 2.6–5.2 μm (x̄ = 3.9 ± 0.5 μm, n = 30) diam., hyaline, slightly loose in the center, becoming well-organized, parallel and strongly dense near the ectal excipulum, narrow hyphae 1.3–3.2 μm (x̄ = 2.3 ± 0.4 μm, n = 30) diam., non-gelatinous. Ectal excipulum well-differentiated from the medullary part, the inner layers generally consists of 5–6 layers *textura angularis* cells, 27–68 μm (x̄ = 42 ± 9 μm, n = 37) thick, cells 6.6–14.4 μm (x̄ = 10.2 ± 2.4 μm, n = 100) diam., wall moderately thick 0.41–0.84 μm (x̄ = 0.6 ± 0.1 μm, n = 50); the outer layers partially uneven proliferous to some gradually smaller brown cells, stack into short and broad triangles to trapezoids, 20–46(–63) μm (x̄ = 31 ± 12 μm, n = 20) thick (excluding the inner layers), cells 4.5–10.2 μm (x̄ = 7.8 ± 1.6 μm, n = 80) diam., wall moderate; cells from the outer inward the inner layers gradually increase in diameter, brown to pale yellow; terminal cells at the margin stretch to elongated *textura prismatica* cells 10–13 μm × 3.3–4.1 μm with rounded ends, wall moderately thick, brown, non-gelatinous. Paraphyses 1.7–2.7 μm (x̄ = 2.3 ± 0.2 μm, n = 35), hyaline, filiform, rounded apex, 2-septate at the middle, unbranched, conspicuous contents not observed, scarcely extending beyond the asci. Asci (91–)109–122 × 8.2–11.5 μm (x̄ = 113 ± 5 × 9.5 ± 0.7 μm, n = 25), unitunicate, 8-spored, almost filling the whole asci, clavate, rounded apex with an amyloid apical pore in Melzer’s reagent, wall incrassated at the apex, 5.5–6.8 μm wide × 2.3–4.2 μm high (x̄ = 6.0 ± 0.5 × 3.2 ± 0.4 μm, n = 25), slightly constricted downward when developing, tapering to a cylindric and aporhynchous subtruncate base, croziers present. Ascospore 14.6–20.4(–22.5) × 4.9–6.2 μm (x̄ = 17.1 ± 1.7 × 5.4 ± 0.4 μm, n = 60), Q = 2.4–3.7(–4.2), Qm = 3.1 ± 0.1, overlapping uniseriate, slightly narrow ellipsoidal with a large guttule, ends rounded at the base, slightly pointed at the apex, slightly curved on the lateral view, hyaline, thin-walled, smooth and aseptate. ***Asexual morph***: Undetermined.

**Figure 6.**
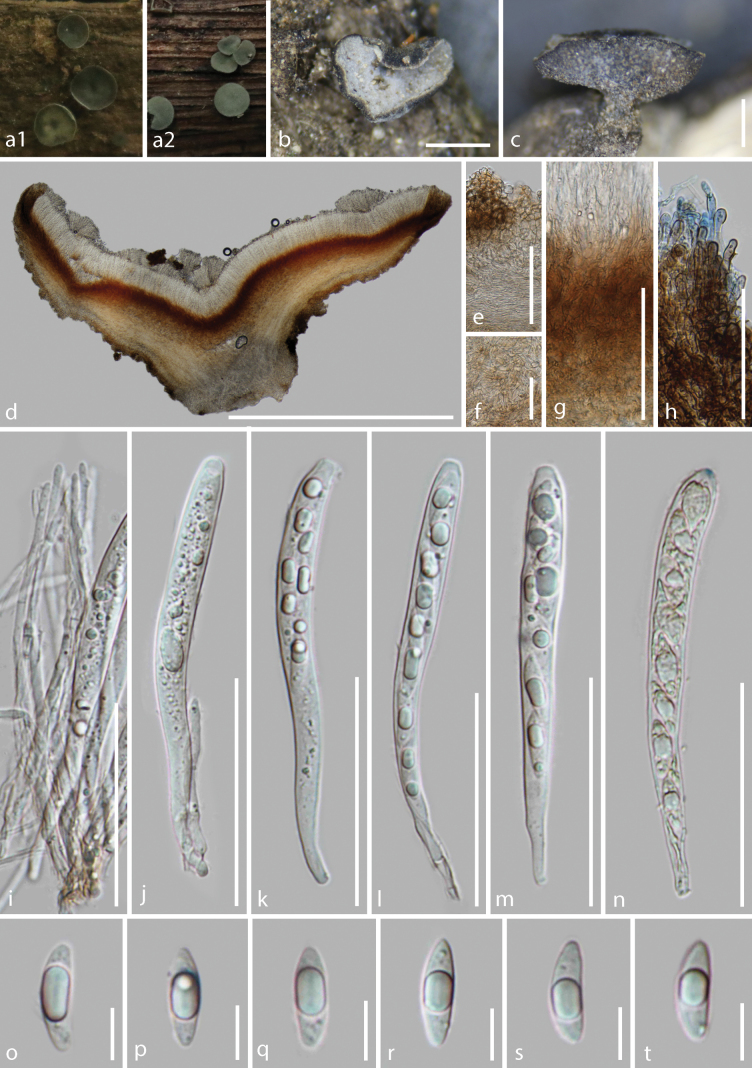
*Tatraeagriseoturcoisina* (HKAS 128276, holotype) **a** fresh ascomata on the wood **d–g** dried ascomata on the wood **d** vertical section of an ascoma **e–h** excipulum **i** paraphyses **j–n** asci (**o–n** asci in Meltzer’s reagent) **o–t** ascospores. Scale bars: 900 μm (**b**); 600 μm (**c**); 1000 μm (**d**); 80 μm (**e, g**); 40 μm (**f**); 60 μm (**h–n**); 10 μm (**o–t**).

##### Material examined.

China, Yunnan Province, Xishuangbanna City, Menghai County, altitude 1660 m, on decayed oak tree branches in a managed plantation, 8 September 2022, Cuijinyi Li, LCJY-1402 (HKAS 128276, holotype); *ibid*., Menghai County, altitude 1500 m, on decayed oak tree branches in a managed plantation, 8 September 2022, Cuijinyi Li, 22-9-8-5 (HKAS 128277, paratype).

##### Notes.

The distinctive characteristics of *Tatraeagriseoturcoisina* are greyish-green apothecia, with yellowish-white to snow white discs when dry, narrow hyphae of medullary excipulum, short aporhynchous asci and slightly narrow ellipsoidal ascospores without septa.

Phylogenetically, *T.griseoturcoisina* grouped with *T.clepsydriformis* with 85% ML bootstrap support and 0.96 Bayesian probability in the combined LSU and ITS phylogeny (Fig. 2). A pairwise homoplasy index (PHI) test was conducted using a five-gene dataset (ITS, LSU, mtSSU, RPB1 and RPB2) to assess the recombination level between clades of *T.griseoturcoisina* and *T.clepsydriformis*. The results revealed that there were no significant recombination events observed between these two groups (Фw > 0.05), indicating that they are genetically isolated and thus supporting them as distinct species (Fig. 3). *Tatraeagriseoturcoisina* is distinct from all other species based on its unique macro-characteristics of greyish-green apothecia, dried discs and receptacles. Micro-characteristics of *T.griseoturcoisina* resemble *T.clepsydriformis* by having narrow hyphae of medullary excipulum, short asci and smaller ellipsoidal ascospores, but it is distinct from *T.clepsydriformis* by having a thinner medullary excipulum (164–308 μm *vs.*335–535 μm), longer (17.1 × 5.4 μm *vs.* 15.2 × 5.7 μm) and curved ascospores. *Tatraeagriseoturcoisina* can be distinguished from the other five species (*T.aseptata*, *T.dumbirensis*, *T.macrospora*, *T.yunnanensis* and *T.yuxiensis*) based on its short asci (109–122 μm) and ascospores (14.6–20.4 μm) (see Suppl. material 1).

#### ﻿ Tatraea yunnanensis

Taxon classificationFungiHelotialesHelotiaceae

C.J.Y. Li & Q. Zhao sp. nov.

C3E5A6FA-7373-5CDE-B6E3-198EF12D885F

Index Fungorum: IF901180

Facesoffungi Number: Fo15192

Fig. 7

##### Etymology.

The specific epithet refers to the locality from where the type species was collected.

##### Holotype.

HKAS 128273.

##### Description.

Saprobic on the decayed wood. ***Sexual morph***: Apothecia 3.8–5.0 mm wide × 2.5–4.1 mm high (x̄ = 4.8 ± 0.8 × 3.7 ± 0.8 mm, n = 10) when dry, scattered, superficial when fresh, short stipitate, glabrous. Disc circular, flat or slightly concave when fresh, yellowish white (4A2) to orange white (5A2), edge strongly curl inward towards the disc when dry, pastel green (29A4-30A4) to light green (29A5), dull green (29E4) near the edge. Margins concolorous to the disc when fresh, white or concolorous to the disc when dry. Receptacle rough and pale greyish orange (5B3) with loose, finely yellowish brown (5E8) pustules when fresh, rough and light brown (5E4) with finely dark pustules and irregular patches when dry, center of vertical section appears white powder, outwardly yellowish waxy materials. Stipe 0.5 mm wide × 1.1 mm long, concolorous to the receptacle, dense finely granular pustules. Hymenium 173–213 μm (x̄ = 192 ± 10 μm, n = 20) thick, hyaline. Subhymenium 51.5–68.5 μm (x̄ = 60.5 ± 5.0 μm, n = 27) thick, slightly indistinguishable from the medullary excipulum, comprised of dense and unordered brown (5D4) hyphae of *textura intricata*, hyphae 2.2–5.4 μm (x̄ = 3.9 ± 0.9 μm, n = 20) diam., with excipulum at the margin. Medullary excipulum 435–560 μm (x̄ = 518 ± 48 μm, n = 10) thick, well-developed, comprised of thin-walled, septate, branched, hyaline and lose hyphae of *textura intricata* in the center, partially cells of hyphae becoming swollen, hyphae 3.6–7.8(–8.9) μm (x̄ = 5.3 ± 0.9 μm, n = 77) diam., becoming well-organized, parallel near to the ectal excipulum, hyphae narrower, non-gelatinous. Ectal excipulum of the inner layers usually comprised of 3–5 layers of *textura angularis* to *textura prismatica* cells oriented vertically to the receptacle, brown to hyaline from the outside to inside, 37–65 μm (x̄ = 49.5 ± 8.8 μm, n = 70) thick, cells 8.4–16.5 μm (x̄ = 13.0 ± 1.1 μm, n = 50) diam., wall moderately thick 0.56–0.9 μm (x̄ = 0.72 ± 0.11 μm, n = 84) thick; the outer layers uneven dense proliferous 2–10 layers of *textura angularis* to *textura prismatica* cells, 20–74 μm (x̄ = 49 ± 12 μm, n = 43) thick (out of the inner layers), usually parallel to receptacle, forming an inverted arched or irregular shaped, not obvious change in *textura angularis* cells on diameter, slightly larger on *textura prismatica* cells, brown; terminal cells at the margin, indistinctively elongated. Paraphyses 1.5–2.8 μm (x̄ = 2.1 ± 0.4 μm, n = 80) wide, hyaline, filiform, rounded apex, 3–4-septate, sometimes branched at mid and base, with conspicuous contents and fine oil drops, scarcely extending beyond the asci. Asci (163.8–)170.9–197.4 × 10.1–15.5 μm (x̄ = 180.5 ± 7.2 × 12.6 ± 1.4 μm, n = 50), unitunicate, 8-spored, almost filling in some short asci, cylindric or subclavate, rounded apex with an amyloid apical pore in Melzer’s reagent, apical wall incrassated, 6.4–9.3(–10.3) × 2.7–5.1(–6.0) (x̄ = 7.8 ± 0.8 × 3.8 ± 0.7 μm, n = 40), thicken when immature, tapering to subtruncated base, croziers present. Ascospore 32.5–42.4 × 4.8–7.1 μm (x̄ = 36.3 ± 2.6 × 6.5 ± 0.4 μm, n = 50), Q = 4.4–6.5(–7.2), Qm = 5.6 ± 0.4, uniseriate or overlapping uniseriate, elongated to narrow fusiform with 1–2 guttules to multiple granules, hyaline, slightly curved, bluntly rounded at the base, slightly pointed at the apex, thin-walled, smooth and aseptate. ***Asexual morph***: Undetermined.

**Figure 7.**
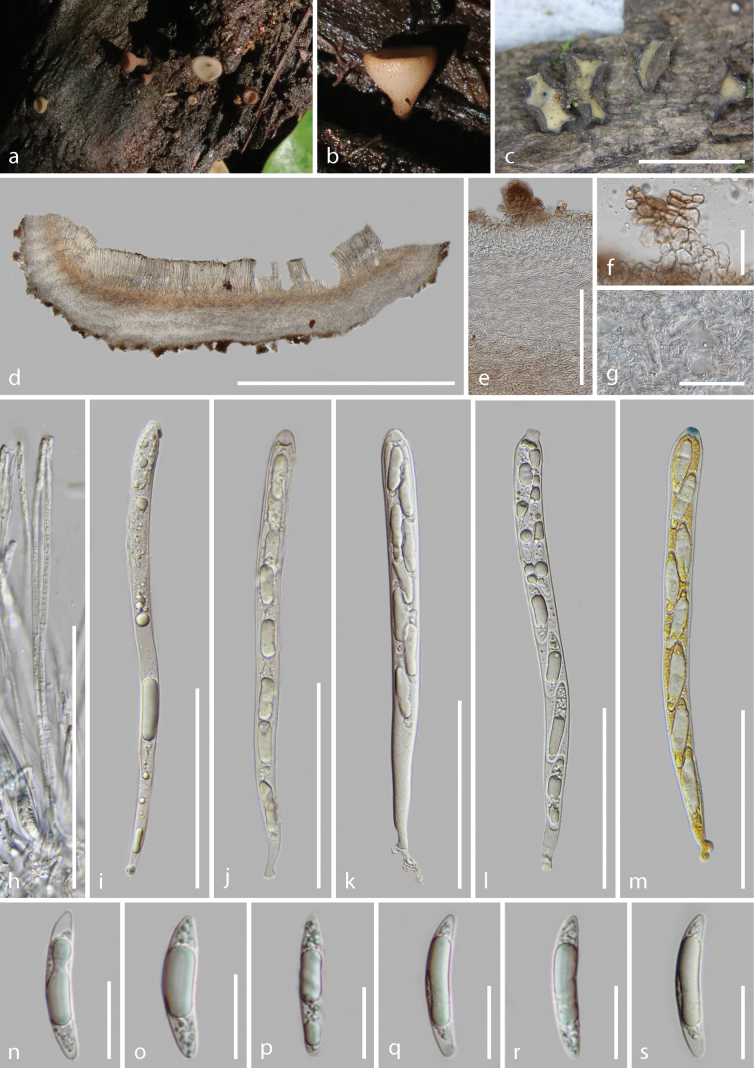
*Tatraeayunnanensis* (HKAS 128273, holotype) **a–b** fresh ascomata on the wood **c** dried ascomata on the wood **d** vertical section of an ascoma **e–g** excipulum **h** paraphyses **i–m** asci (**m** asci in Meltzer’s reagent) **n–s** ascospores. Scale bars: 3.5 mm (**c**); 1200 μm (**d**); 300 μm (**e**); 50 μm (**f**); 100 μm (**g**); 80 μm (**h–m**); 20 μm (**n–s**).

##### Material examined.

China, Yunnan Province, Puer City, Jingdong County, altitude 1455 m, on the decayed wood, 23 August 2022, Cuijinyi Li, LCJY-1218 (HKAS 128273, holotype); *ibid*., Tengchong City, altitude 1714 m, on decayed wood, 16 August 2022, Cuijinyi Li, LCJY-1119-2 (HKAS 128272, paratype).

##### Notes.

The distinctive characteristics of *T.yunnanensis* are large (4.8 mm wide), brown apothecia with pastel green to light green discs and short stipes, thick medullary excipulum comprising 2–10 layers of inverted proliferous cells, arched or irregular shaped, pleurorhynchous asci with J+ pores, filiform, 3-septate paraphyses and elongated fusiform ascospores without septa.

Phylogenetically, our collections clustered sister to *T.macrospora* with 98% ML bootstrap support and 1.0 Bayesian probability in the combined LSU and ITS phylogeny (Fig. 2). It was shown that the two species do not have any genetic recombination (*Φw* = 1.0) based on the pairwise homoplasy index (PHI) value (Fig. 3). *Tatraeayunnanensis* resembles *T.macrospora* in having cupulate apothecia, yellowish-white to orange-white discs when fresh and large ascospores. In contrast, our species differ from *T.macrospora* by having longer ascospores (32.5–42.4 μm vs. 22-40 μm) with 3–8-septate ascospores (Baral et al. 1999). For *T.macrospora*, the morphological descriptions provided in previous studies are incomplete and lack details on the apothecial size and color, stalk and spores.

#### ﻿ Tatraea yuxiensis

Taxon classificationFungiHelotialesHelotiaceae

C.J.Y. Li & Q. Zhao sp. nov.

A38B8F70-6AC1-5B54-B9AE-A7C169878504

Index Fungorum: IF901187

Facesoffungi Number: Fo15193

Fig. 8

##### Etymology.

The specific epithet refers to the locality from where the type species was collected.

##### Holotype.

HKAS 128268.

##### Description.

Saprobic on the decayed wood. ***Sexual morph***: Apothecia 2.3–4.2 mm wide (x̄ = 3.0 ± 0.6 mm, n = 15) when fresh, 1.2–2.0(–2.5) mm wide × 0.47–0.72 mm high (x̄ = 1.6 ± 0.3 × 0.58 ± 0.09 mm, n = 18) when dry, scattered or gregarious, disk-like with short stipitate, glabrous, developing on the surface of the substrate. Disc flat and circular, slightly convex at the center and downward at the edge when fresh, edge slightly curls inward towards the center, edge of large ascoma somewhat undulating, orange grey (5B2) to brownish grey (5C2) when fresh, dark brownish black or deep green (1D8) to olive (1E8) when dry. Margins concolorous to the disc when fresh, white to pale yellow or concolorous to the receptacles when dry. Receptacle dark brownish black or dark ochraceous-brown when dry, slightly rough and finely pustules when mature. Stipe 270–480(–650) μm wide × 190–400 μm long (x̄ = 400 ± 86 × 320 ± 76 μm, n = 18), short, regular cylindrical, concolorous to the receptacle or nearly black, almost smooth on the surface. Hymenium 173–227 μm (x̄ = 210 ± 17 μm, n = 15), hyaline. Subhymenium not obvious. Medullary excipulum 273–330 μm (x̄ = 307 ± 22 μm, n = 15) thick, well-developed, comprised of thin-walled, septate, pale brown and slightly loose hyphae of *textura intricata*, hyphae (3.5–)4.2–8.7 μm (x̄ = 6.2 ± 1.5 μm, n = 70) diam., hyaline, becoming dense near to the hymenium, darkening, dense and well-organized parallel near to the ectal excipulum, non-gelatinous. Ectal excipulum of the inner layers generally comprised of 3–6 layers vertically oriented *textura angularis* cells, 34–62(–77) μm (x̄ = 49 ± 10 μm, n = 60) thick, pale brown to hyaline toward inwards, cells of inner layers 10.5–18.5(–20.4) μm (x̄ = 14.0 ± 2.6 μm, n = 100) diam., wall (0.49–)0.65–1.09(–1.3) μm (x̄ = 0.87 ± 0.15 μm, n = 100) thick; proliferate 3–4 layers irregular-shaped and minimal *textura angularis* or *textura prismatica* cells, 4.0–8.4 μm (x̄ = 6.3 ± 1.3 μm, n = 100) diam., wall 0.59–0.94 μm (x̄ = 0.77 ± 0.12 μm, n = 70) thick; terminal cells at margin obviously elongated to 17–33 μm × 3.6–5.9 μm, slightly curved and soft, apex rounded and sometimes swollen, thin-walled, pale brown or hyaline. Paraphyses 1.9–3.3 μm (x̄ = 2.6 ± 0.5 μm, n = 70) wide, hyaline, filiform, rounded apex, 1–2-septate, unbranched, with conspicuous contents in Melzer’s reagent, scarcely extending beyond the asci. Asci (159.6–)167.4–190.7(–200.0) × 11.3–15.8 μm (x̄ = 177.5 ± 8.7 × 13.1 ± 1.2 μm, n = 30), unitunicate, 8-spored, cylindric or subclavate, apically rounded with an amyloid apical pore in Melzer’s reagent, thickened wall at apex, 7.2–11.5 × 3.0–5.7 (x̄ = 9.2 ± 1.5 × 4.2 ± 0.7 μm, n = 40), tapering to a pleurorhynchous subtruncate base, croziers present. Ascospore (24.1–)26.2–34.9(–36.5) × (6.3–)7.0–8.9(–9.5) μm (x̄ = 30.0 ± 2.8 × 7.9 ± 0.6 μm, n = 100), Q = (2.8–)3.2–4.6(–5.2), Qm = 3.8 ± 0.2, uniseriate, elongated ellipsoidal with a large guttule, slightly curved and asymmetrical on the lateral view, ends rounded, hyaline, thin-walled, smooth, aseptate, appearing 1-septate when germinating. ***Asexual morph***: Undetermined.

**Figure 8.**
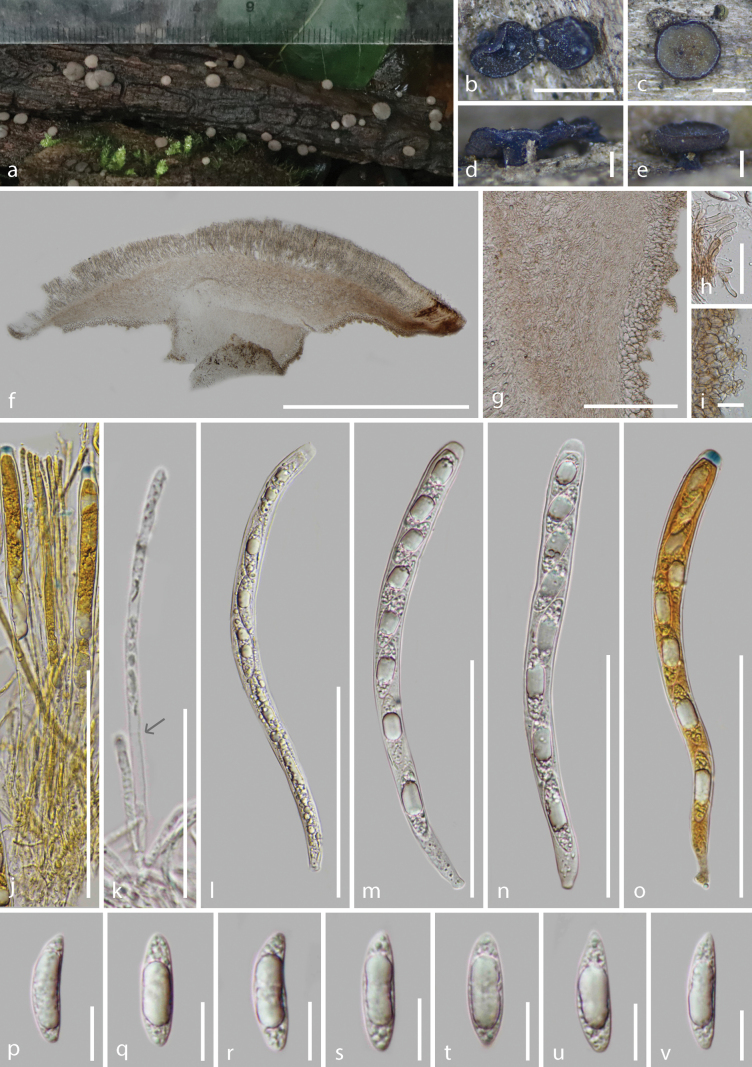
*Tatraeayuxiensis* (HKAS 128268, holotype) **a** fresh ascomata on the wood **b–e** dried ascomata on the wood **f** vertical section of an ascoma **g–i** excipulum **j–k** paraphyses **l–o** asci (**o–p** asci in Meltzer’s reagent) **p–v** ascospores. Scale bars: 1.5 mm (**b**); 700 μm (**d–c**); 400 μm (**e**); 800 μm (**f–g**); 1000 μm (**h**); 200 μm (**i**); 100 μm (**j**); 70 μm (**k–p**); 15 μm (**q–v**).

##### Material examined.

China, Yunnan Province, Yuxi City, Xinping County, altitude 2090 m, on soft decayed unknown wood in managed plantation, 5 June 2022, Cuijinyi Li, LCJY-633 (HKAS 128268, holotype); *ibid*., altitude 2340 m, on decayed unknown wood, 6 June 2022, Cuijinyi Li, LCJY-634 (HKAS 128270, paratype).

##### Notes.

The distinctive characteristics of *T.yuxiensis* are orange-grey to brownish-grey discs when fresh, dark brownish-black or dark ochraceous-brown when dry, short and regular cylindrical stipe, 3–4 layers of proliferous minimal cells, pleurorhynchous asci and elongated ellipsoidal and laterally asymmetrical ascospores.

Morphologically, *T.yuxiensis* resembles *T.aseptata* with their similar-sized and disk-like apothecia with a short stipe, filiform paraphyses, cylindric asci and ellipsoidal ascospores. In contrast to *T.aseptata*, *T.yuxiensis* has darker receptacles, regular cylindrical stipes, inconspicuous subhymenium, thicker medullary excipulum (273–330 μm *vs.* 120–145 μm) with larger terminal cells (17–33 μm × 3.6–5.9 μm *vs.* 13.1–15.3 μm × 2.6–3.8 μm) that almost appear as 3–4 layers of proliferous minimal cells and ascospores with higher length-width ratio (3.8 vs. 2.97). Furthermore, our collections of *T.aseptata* (HKAS 128265, HKAS 128269, HKAS 128271, HKAS 128274) have shorter asci (136.7–157.8 μm vs. 167.4–190.7 μm) and 8–12 layers of proliferous cells, compared to *T.yuxiensis*.

Phylogenetically, *T.yuxiensis* clustered sister to *T.aseptata* with 100% ML bootstrap support and 1.0 Bayesian probability in the combined LSU and ITS phylogeny (Fig. 2). The pairwise homoplasy index (PHI) indicated no significant genetic recombination (*Φw* = 1.0) between *T.yuxiensis* and *T.aseptata* and confirmed that they are different species (Fig. 3).

## ﻿Discussion

*Tatraea* was initially collected a 100 years ago from the Nizke Tatra Mountains in Europe and was subsequently found in several countries (Velenovský 1934; Baral et al. 1999). In Britain and Croatia, *T.dumbirensis* is listed on the Red List as a threatened species (Ujházy et al. 2018). *Tatraeamacrospora* appeared in some countries, but no official records were found. Therefore, the accuracy of species identification could not be confirmed. Since the study in 1999, there have been no new species reports in *Tatraea* from other continents. All collections in this study were collected from Yunnan, China, of which most were collected from protected natural forests and from areas comprising mainly oak trees. Some species are found in plantations that have been protected and nursed for many years, however, the host is too decayed to identify. In most cases, the decayed oak wood is still the main nutrient provider in forests. After the addition of our collections (*T.clepsydriformis* and *T.griseoturcoisina*), the description of *Tatraea* should be extended from long asci and large ascospores to include slightly shorter asci and smaller spores, as well as the initial greyish turquoise color of the apothecia.

In the past, the type species, *T.dumbirensis* was incorrectly recognized to be a member of the Leotiaceae or Sclerotiniaceae (Velenovský 1934; Baral et al. 1999). The exclusion from the Sclerotiniaceae was due to the absence of darkening and sclerotia formation in the cultures (Baral et al. 1999). In the present taxonomic study, *Tatraea* was included in Helotiaceae, and we also agreed on this treatment based on the ITS analysis in our study (Vasilyeva 2010). The genus-level placements of each species in *Tatraea* changed after adding data from other genera into the analysis. Previously, *T.clepsydriformis* and *T.griseoturcoisina* clustered into separate clades, later clustered into a single main clade as sister sub-clades after adding more taxa, and their micro-morphological characteristics were more similar. In the phylogenetic analyses, the taxonomic status of species is provisional due to the lack of genetic information for the type species. To assess the significant recombination levels of related species, we performed five gene analyses individually and for the combined dataset, both of which provided evidence for them being different species. The dilemma for conducting research is the paucity of available molecular information for the known species. More informative loci were provided in this study, including mitochondrial genes and protein genes, hence, future taxonomy, phylogeny research and evolutionary studies in Helotiaceae can be benefited from this study. Additionally, some species with similar morphological characteristics to *Tatraea*, such as *Ciboriafusispora*, are currently unable to transfer due to a lack of evidence and fresh samples. Therefore, more research with more fresh specimens is essential to facilitate the classification of these species.

## Supplementary Material

XML Treatment for Tatraea aseptata

XML Treatment for Tatraea clepsydriformis

XML Treatment for Tatraea griseoturcoisina

XML Treatment for Tatraea yunnanensis

XML Treatment for Tatraea yuxiensis

## ﻿Additional information

### Author ORCIDs

Cui-Jin-Yi Li https://orcid.org/0000-0002-2805-7071

Kandawatte Wedaralalage Thilini Chethana https://orcid.org/0000-0002-5816-9269

Prapassorn Damrongkool Eungwanichayapant https://orcid.org/0000-0001-8005-4137

De-Qun Zhou https://orcid.org/0009-0009-3459-3186

Qi Zhao https://orcid.org/0000-0001-8169-0573

### Data availability

All of the data that support the findings of this study are available in the main text or Supplementary Information.

## Supplementary materials

Supplementary material 1 Main differences between Tatraea Data type docx https://binary.pensoft.net/file/983955This dataset is made available under the Open Database License (http://opendatacommons.org/licenses/odbl/1.0/). The Open Database License (ODbL) is a license agreement intended to allow users to freely share, modify, and use this Dataset while maintaining this same freedom for others, provided that the original source and author(s) are credited.Author: Cui-Jin-Yi Li
