# Supplementary material for: ﻿Additional four species of Tatraea (Leotiomycetes, Helotiales) in Yunnan Province, China
Source: MycoKeys. 2024 Feb 14;102:127–54. doi: 10.3897/mycokeys.102.112565 (PMC10882549; doi:10.3897/mycokeys.102.112565)
Supplement: Supplementary material 1 — Main differences between Tatraea [file mycokeys-102-127-s001.docx]

**Appendix 1** Main differences between *Tatraea*.

| **Characters Taxon name** | ***T. aseptata*** | ***T. clepsydriformis*** | ***T. dumbirensis*** | ***T. griseoturcoisina*** | ***T. macrospora*** | ***T. yunnanensis*** | ***T. yuxiensis*** |
| --- | --- | --- | --- | --- | --- | --- | --- |
| **Apothecia** | †2.5–4.7;  #1–2.4 × 0.6–1.2;  Discoid | †1.3–3.5;  #0.9–1.3 × 0.6–0.9;  Hourglass | (l-)2-6(-8) × 1-4;  Flat cupulate | †2.5–4.0；  #1.0–2.1 × 0.6–0.8  Discoid | Whitish | #3.8–5.0 × 2.5–4.1;  Cupulate | †2.3–4.2  #1.2–2.0 × 0.47–0.72;  Disk-like |
| **Disc** | †Light brown to slightly dark alabaster grey;  #Dark brown to dull green or greyish green | †Pale grey;  #Pale orange near the center, darken near the edge; | †Pale greyish to cream-brownish-grey；  #Ochraceous to dark brown | †Greyish turquoise;  #Yellowish white to snow white |  | †Yellowish white to orange white;  #Pastel green to light green, dull green near the edge | †Orange grey to brownish grey;  #Dark brownish black or deep green to olive |
| **Receptacle** | †Finely pustulates, smooth and brown;  #Yellowish brown, flank darker | †Dark yellowish brown;  #Light brown to hazel brown |  | #Finely pustulates, dark brown nearly black, with slightly darken and irregular veins on the surface | externally furfuraceous | †Finely pustulates, pale greyish orange;  #Light brown with irregular patches, outwardly yellowish waxy materials | #Finely pustulates, dark brownish;  black or dark ochraceous-brown |
| **Stipe** | #280–725 × 340–735;  †Taper from top to bottom;  #light brown, ridged and rough | #360–596 × 463–571;  †concolorous to the receptacle or pale yellow;  #butter yellow, slightly rough | 0.9-2 × 0.8-2;  cylindrical or slightly tapering, pale brownish-grey, ochraceous brown to brown | #330–360 × 220–440;  #concolorous to the receptacle, rough |  | 0.5 × 1.1;  #concolorous to the receptacle, rough | #270–480 × 190–400;  #regular cylindrical, concolorous to the receptacle or nearly black, almost smooth |
| **Hymenium** | 142–190 | 122–155 |  | 103–138 |  | 173–213 | 173–227 |
| **Subhymenium** | 35–52;  Brown hyphae, hyphae wide 3.4–4.3 | (24–)36–60(–65);  Golden brown, hyphae wide 2.2–2.9 | Indistinguishable | 43–66;  Brownish orange, hyphae wide 1.3–2.9(–3.9) |  | 51.5–68.5;  Slightly indistinguishable, brown, hyphae wide 2.2–5.4 | Indistinguishable |
| **Medullary excipulum** | 120–145;  Very pale brown, hyphae wide 4.3–7.4 | 335–535;  Pale brown, hyphae wide 3.3–5.1 | 750–950;  Hyaline, hyphae wide 2.3–7.6 | 164–308;  Pale brown to pale yellow, hyphae wide 2.6–5.2, narrow hyphae wide 1.3–3.2 |  | 435–560;  Pale brown, hyphae wide 3.6–7.8(–8.9) | 273–330;  Pale brown, hyphae wide (3.5–)4.2–8.7 |
| **Ectal excipulum** | ‡3–4 layers, 30–48;  §8–12 layers, stack into triangles to trapezoids, 50–89 (including the inner layers);  ¶3–4 layers at the flank stretch to 13.1–15.3 × 2.6–3.8, straight, ends narrow and slightly sharp | ‡3–5 layers, 29–80;  ¶Inconspicuous elongated | ‡30–75; | ‡5–6 layers, 27–68;  §Stack into triangles to trapezoids, 20–46 (excluding the inner layers);  ¶Stretch to elongated, cells 10–13 × 3.3–4.1, ends rounded |  | ‡3–5 layers, 37–65;  §Dense 2-10 layers, 20–74 (out of the inner layers), inverted arched or irregular shape;  ¶Indistinctive elongated | ‡3–6 layers, 34–62(–77);  §3–4 layers, irregular-shaped;  ¶Elongated to 17–33 × 3.6–5.9, slightly curved and soft, apex rounded and sometimes swollen |
| **Paraphyses** | 1.9–3.7;  0–2 septate, unbranched, observed conspicuous lipid bodies, scarcely extending beyond the asci | 2.1–3.4;  1–3 septate, unbranched, not obviously conspicuous contents, scarcely extending beyond the asci | 2 5-3 5;  Distantly septate, sometimes branched, observed conspicuous lipid bodies, slightly shorter than living asci | 1.7–2.7;  2 septate in the middle, unbranched, conspicuous contents not observed, scarcely extending beyond the asci |  | 1.5–2.8;  3–4 septate, sometimes branched, observed conspicuous contents with some fine oil drops, scarcely extending beyond the asci | 1.9–3.3;  1–2 septate, unbranched, observed conspicuous contents, scarcely extending beyond the asci |
| **Asci** | 136.7–157.8 ×13.2–16.0; Clavate, slightly curved, obconical or short subtruncate base | 112.4–135.8 × 8.2–12.2; Cylindric or subclavate, cylindric and aporhynchous subtruncate base | 180–208 × 12.5–15.8;  Cylindrical | 109–122 × 8.2–11.5;  Clavate, cylindric and aporhynchous subtruncate base | l55–200 × 11–19; | 170.9–197.4 × 10.1–15.5; Cylindric or subclavate, subtruncate base | 167.4–190.7 × 11.3–15.8;  Cylindric or subclavate, pleurorhynchous subtruncate base |
| **Ascospores** | 24.6–31.6 ×7.8–10.0, Q = 2.5–3.7, Qm = 3.1 ± 0.1;  Reniform with a large guttule and several multiple granulates, slightly asymmetrical, aseptate | 14.0–17.9 × 5.1–6.8, Q = 2.1–3.2, Qm = 2.7 ± 0.1;  Ellipsoidal with a large guttule, almost symmetrical, aseptate | 17–23 × 5.8–7.5, 15–20 × 5–7;  Fusoid ellipsoid, reniform, densely multiguttulate when living, one, two or more large guttules when dead, 1(–2)-septate | 14.6–20.4 × 4.9–6.2, Q = 2.4–3.7, Qm = 3.1 ± 0.1;  Slightly narrow ellipsoidal with a large guttule, slightly asymmetrical, aseptate | 22–40 × 6–8;  oblong, 4-8 large LBs when Living, finally 3–8-septate, budding off globose conidia on short tubules | 32.5–42.4 × 4.8–7.1; Q = 4.4–6.5, Qm = 5.6 ± 0.4;  Elongated narrow fusiform, 1–2 guttule and multiple granulates, slightly curved, aseptate | 26.2–34.9 ×7.0–8.9; Q = 3.2–4.6, Qm = 3.8 ± 0.2;  Elongated ellipsoidal with a large guttule aseptate, appearing 1-septate when germinating tubes |

Notes: ‘†’ indicates fresh state; ‘#’ indicates dry state; ‘‡’ indicate inner layers; ‘§’ indicates partially uneven proliferous layers; ‘¶’ indicates terminal cells; values of apothecia and stipe indicate ‘wide × high’, units of apothecia and some stipes (*T. dumbirensis* and *T. macrospora*) are millimeters (mm), and others units are micrometer (μm).
